# A taxonomic contribution to the genus *Dolichomitus* Smith (Hymenoptera, Ichneumonidae, Pimplinae) from Brazil

**DOI:** 10.3897/zookeys.221.3558

**Published:** 2012-09-14

**Authors:** Ana Paula da Silva Loffredo, Angélica Maria Penteado-Dias

**Affiliations:** 1 Programa de Pós Graduação em Ecologia e Recursos Naturais, Universidade Federal de São Carlos – UFSCar, CP 13565–905, São Carlos, SP, Brazil; 2 Departamento de Ecologia e Biologia Evolutiva, Universidade Federal de São Carlos – UFSCar, CP 13565–905, São Carlos, SP, Brazil

**Keywords:** Neotropical, new species, savannah, distribution

## Abstract

In the present study, two new species of Pimplinae, *Dolichomitus jatai* **sp. n.** and *Dolichomitus moacyri* **sp. n.** are described, and the distribution range of *Dolichomitus annulicornis* (Cameron, 1886) is extended. The specimens were collected using Malaise traps in areas of Atlantic forest and Brazilian savannah (cerrado) in southeastern Brazil and are deposited in a Brazilian collection (DCBU).

## Introduction

*Dolichomitus* Smith, 1877 includes 72 species, with 13 recorded from the Neotropics (Gauld et al. 1998; Yu et al. 2005), of which four occur in Brazil: *Dolichomitus annulicornis* (Cameron, 1886), *Dolichomitus megalourus* (Morley, 1914) and the new species *Dolichomitus jatai* sp. n. and *Dolichomitus moacyri* sp. n. This genus is not monophyletic, but some of the component species groups are monophyletic lineages (Gauld et al. 2002). Specimens of *Dolichomitus* are characterized by oblique grooves anterolaterally, which form triangular areas on tergite II of the metasoma, and the lateral expansion of the lower valve of ovipositor. Townes (1975) published a description of the Hymenoptera with the longest ovipositors, describing the neotropical species *Dolichomitus bivittatus* and *Dolichomitus hypermeces*. Gauld et al. (1998) grouped eight species from Costa Rica into four groups: *longicauda*, *taeniatus*, *irritator* and *zonatus*. Nothing is known about the biology of these groups of species.The purpose of the present paper is to describe the two new species and to update the known distribution of *Dolichomitus annulicornis*.

## Material and methods

The examined specimens were collected using Malaise traps in different localities of southeastern Brazil and are deposited in the DCBU collection (Departamento de Ecologia e Biologia Evolutiva, Universidade Federal de São Carlos, São Carlos, SP, Brazil). The terminology used mostly follows Gauld (1991) and Gauld et al. (1998). Color pictures were taken with a Leica stereomicroscope with LAS software; grayscale pictures were taken with an FEI Quanta 250 SEM under low-vacuum conditions.

## Results

Gauld (1991) reported the distribution of *Dolichomitus annulicornis* as occurring from southern Mexico southward to equatorial South America. Adding to the material from the Natural History Museum of London identified by I. Gauld and reports found in the literature, we identified one male and three female specimens of *Dolichomitus annulicornis* collected in southeastern Brazil, expanding the distribution of this species southward. These new specimens also constitute the first records of this species in seasonal dry forests (Gauld 1991). We collected *Dolichomitus annulicornis* in both semideciduous seasonal forest and dry savannah (cerrado). In addition, the morphological features of specimens of two new species of *Dolichomitus* are described and discussed below.

### Dolichomitus jatai sp. n.

urn:lsid:zoobank.org:act:D9531345-3F6C-4394-A950-D48544A334D8

http://species-id.net/wiki/Dolichomitus_jatai

Figures 1–2 7–13

#### Material examined.

Type locality. Brazil, SP, Luís Antônio, Estação Ecológica de Jataí, 21°35'16.7"S, 47°47'43.9"W; 15.X.2009; Brazilian savannah, N.W. Perioto and team col., Malaise trap.

#### Type specimen.

Holotype pinned (DCBU): female, Brazil, SP, Luís Antônio, Estação Ecológica de Jataí, Brazilian savannah, 21°35'16.7"S, 47°47'43.9"W, 15.X.2009, Armadilha Malaise II, N.W. Perioto and team col.

Paratypes (DCBU). 1 female, same as the holotype, 16.IX.2009; 2 females and 1 male, same as the holotype, 21°36'10.2"S, 47°46'47.6"W, 16.IX.2009, 27.V.2009 and 03.IX.2008, respectively; 1 male, Brazil, SP, Macaubal, 20°44'34"S, 49°55'45'W, 03.IV.2008, semideciduous seasonal forest, F. Noll col., Malaise trap; 1 male, Brazil, SP, Itirapina, 22°13'09"S, 47°54'04"W, 6.XII.2008, riparian forest, A.M.P. Dias col., Malaise trap; 1 male, Brazil, SP, São Carlos, Fazenda Pinhal, 22°08' 21.80"S, 47°50' 56.57"W, 20.XI.2004, A.M.P. Dias col., Malaise trap.

**Figures 1–6.**
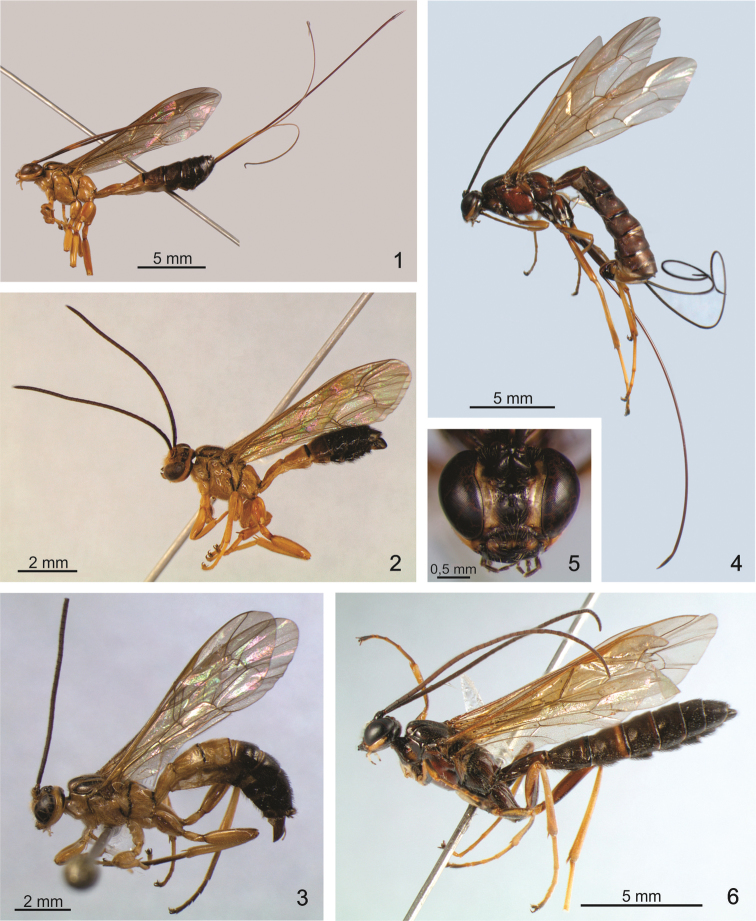
*Dolichomitus jatai* sp. n. female. **1** lateral habitus **2** male, lateral habitus. *Dolichomitus annulicornis* **3** lateral habitus. *Dolichomitus moacyri* sp. n., female **4** lateral habitus **5** head, frontal view **6** male, lateral habitus.

**Figures 7–12.**
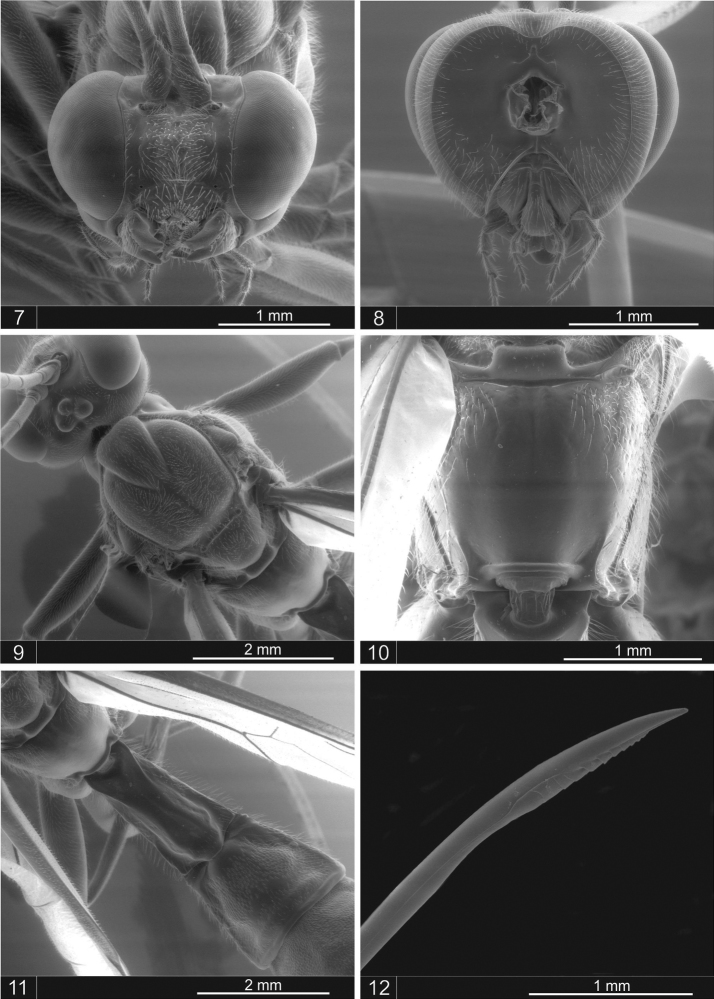
*Dolichomitus jatai* sp. n., female. **7** head, frontal view **8** head, posterior view **9** mesoscutum, dorsal view **10** propodeum, dorsal view **11** tergites I–II **12** ovipositor tip.

Holotype: female (Fig. 1). Body length: 13.7 mm; fore wing length: 9.0 mm. Head (Fig. 7). Antenna with 35 segments, the last flagellomere 2.5x as long as the anterior; mandible with upper tooth more or less equal in length to the lower tooth; clypeus apically bilobate; lower face centrally punctuated with hairs. Occipital carina mid-dorsally dipped; occiput with a mid-dorsal notch (Fig. 8). Anterior margin of pronotum reflexed upward; epomia distinct; mesoscutum with setiferous punctures; notauli very strongly impressed anteriorly (Fig. 9). Mesopleuron smooth and shiny centrally; epicnemial carina very strong ventrally, with a shallow mid-ventral dip; metapleuron punctate with hairs; submetapleural carina complete; propodeum (Fig. 10) dorsally smooth, with setiferous punctures anterolaterally; pleural carina complete.

Metasoma: Tergite I of the metasoma (Fig. 11) with a smooth central area that is defined laterally by carinae convergent posteriorly to the hind margin; tergite II punctuate with hairs, 1.1 times as long as posteriorly broad, with a shallow groove anterolaterally, margin posteriorly smooth and polished; tergites III + with setiferous punctures, posterior margin smooth and polished. Ovipositor 5.6 times the length of the hind tibia, more or less straight (Fig. 12).

Color: Yellow and black or dark brown; head yellow, tips of mandible and occiput black; antenna with scape and pedicel yellow, proximal five flagellomeres black, the 6th and 7th yellow, the remainder brownish; mesoscutum with three black stripes; the anterior margin and posterior lateral margin of the propodeum with a narrow black stripe; pronotum, mesopleuron and metapleuron with a black posterior margin; the posterior margin of tergites II–VI black; tergites III+ dark brown. Legs yellow, fore-femur black, striped dorsally; tips of all tarsal claws brownish yellow. Ovipositor sheath brownish. Wings yellowish; pterostigma brownish.

**Figures 13–18.**
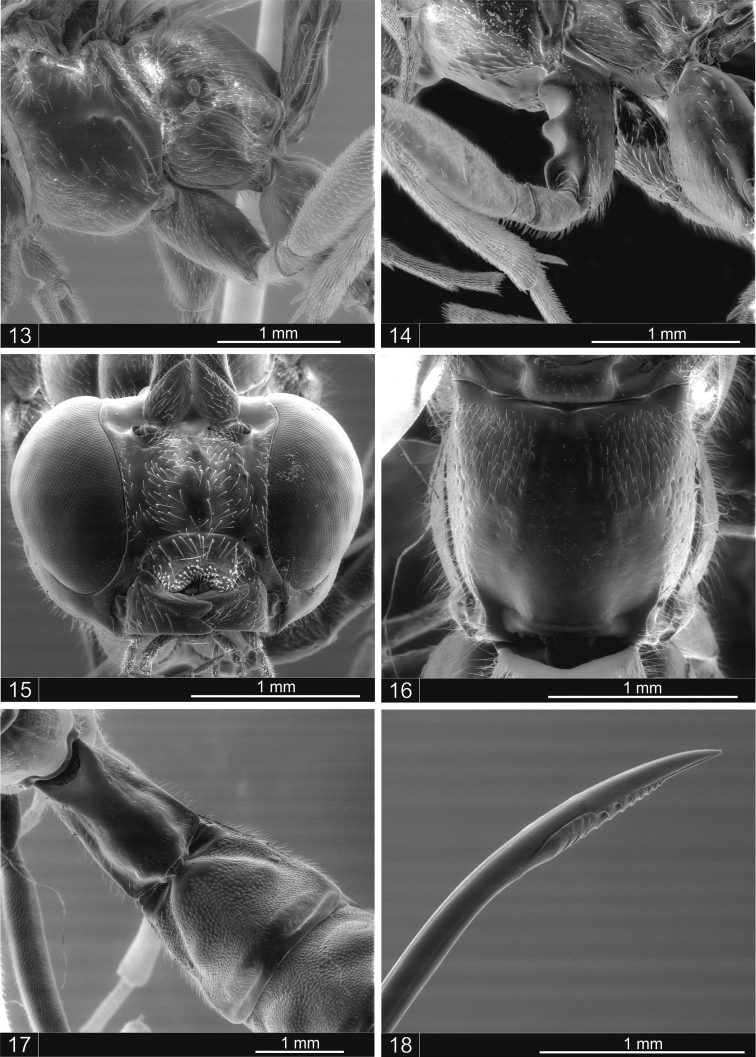
*Dolichomitus jatai* sp. n., male. **13** mid-coxa, lateral view. *Dolichomitus annulicornis*, male**14** mid-coxa, lateral view. *Dolichomitus moacyri* sp. n., female **15** head, frontal view **16** propodeum, dorsal view **17** tergites I–II **18** ovipositor tip.

Male (Fig. 2). Essentially as the female but with body length 10.9 mm; fore wing all yellowish with length 8.6 mm; antenna with 34 segments, the last flagellomere 1.5x as long as the anterior; the proximal four segments brown, the 5th, 6th and 7th slightly yellowish and the remainder brownish; mid-coxa with surface evenly convex (Fig. 13).

#### Etymology.

The name of the species refers to the locality of collection of the material for study.

#### Distribution.

Brazil.

*Dolichomitus jatai* sp. n. seems to belong to the *zonatus* species group, which are vespid mimics and are predominantly yellowish with brown or black marks; the wings are yellowish, and the males have shorter bodies. This species is similar to *Dolichomitus annulicornis* (Fig. 3), differing in the color of the flagellomeres: whereas the proximal five flagellomeres are black and the 6^th^ and 7^th^ yellow in *Dolichomitus jatai* sp. n., the proximal three or four flagellomeres are black and the next four or five whitish yellow in *Dolichomitus annulicornis*. The propodeum is dorsally smooth in *Dolichomitus jatai* sp. n., whereas in *Dolichomitus annulicornis*, there is a smooth and polished area that widens posteriorly. The males of the two species differ in the form of the mid-coxa: in *Dolichomitus annulicornis*, there are prominences separated by deep concavities (Fig. 14), and in *Dolichomitus jatai* sp. n., the surface of the mid-coxa is evenly convex (Fig. 13). In *Dolichomitus zonatus*, the propodeum is similar to that of *Dolichomitus annulicornis*, but it is narrower posteriorly. *Dolichomitus cantillanoi* has a narrower median longitudinal groove and distinct lateromedial secondary furrows, which are never present in *Dolichomitus zonatus*, *Dolichomitus annulicornis* or *Dolichomitus jatai* sp. n. *Dolichomitus bivittatus*, Townes, 1975, and *Dolichomitus hypermeces*, Townes, 1975, are different in color and have longer ovipositors, 12.0× and 21.0× as long as the hind tibia, respectively.

### Dolichomitus moacyri

sp. n.

urn:lsid:zoobank.org:act:B0EC55E4-559B-42CD-982C-011048779C4C

http://species-id.net/wiki/Dolichomitus_moacyri

Figures 4–6 15–18

#### Material examined.

Type locality: Brazil, SP, Poços de Caldas, Sítio da Ferradura, S 21°47'3.4"S, 46°37'22.8"W, 13.X.2006, riparian forest, A.E. de Carvalho col., Malaise trap.

#### Type specimen.

Holotype pinned (DCBU): female, Poços de Caldas, SP, Brazil, Sítio da Ferradura, 21°47'3.4"S, 46°37'22.8"W, riparian forest, 13.X.2006, Malaise trap.

Paratypes (DCBU): 1 female, Brazil, SP, Itapeva, Estação Ecológica de Itapeva, 24°4'10.7"S, 49°4'10"W, 15.IV.2008, Brazilian savannah, A.M.P. Dias col., Malaise trap; 1 male, Brazil, SP, Santa Rita do Passa Quatro, Parque Estadual de Vassununga – Pé-do-gigante, 21°40'56"S, W 47^o^37'13"W, 08.X.2007, riparian forest, A.M.P. Dias col., Malaise trap.

Holotype: female (Fig. 4). Length: 16 mm; fore wing length: 12 mm. Antenna with 37 segments; the last flagellomere 2.5× as long as the anterior; mandible with upper tooth more or less equal in length to the lower tooth; clypeus apically bilobate; lower face (Fig. 15) centrally punctuated; occipital carina mid-dorsally dipped. Epomia distinct; mesoscutum with setiferous punctures; notauli very strongly impressed anteriorly. Mesopleuron mostly centrally smooth and polished, with setiferous punctures; epicnemial carina weak laterally; metapleuron with setiferous punctures; submetapleural carina complete. Propodeum with vestigial lateromedial longitudinal carinae posteriorly divergent with central area smooth, anterolaterally with setiferous punctures (Fig. 16); pleural carinae complete.

Metasoma: Tergite I (Fig. 17) with a smooth central area defined laterally by carinae convergent posteriorly to the hind margin; tergites II punctuate with hairs, 0.8 times as long as posteriorly broad, with a shallow groove anterolaterally; tergites III–V with setiferous punctures and a pair of lateromedial swellings and posterior margin narrow, smooth and polished; tergites VI+ punctate with hairs. Ovipositor 5.0 times the length of the hind tibia, with apex declined (Fig. 18).

Color: Reddish-brown with black and yellow markings. Head yellow with vertex brown; occipital region, central frons and mandible black (Fig. 5); antenna dark brown, three black stripes on mesoscutum; dorselum yellow; propleuron black; tergite I of metasoma brownish, tergite II+ reddish brown; distal tarsomeres dark brown; coxa of first pair of legs black with a whitish spot anteroventrally; trochanter brown, whitish ventrally; trochantelus dark brown; femur and tibia yellow with a black stripe laterally; tarsomeres I–IV yellow, dorsally brown; coxa and trochanter of the second pair of legs dark brown, whitish ventrally; trochantelus brown, femur and tibia yellow with a black stripe laterally; tarsomeres I–IV yellow, dorsally brown; hind leg with coxa orange-brown; trochanter, trochantelus and femur yellow, ventrally brown; tibia and tarsomeres I–II yellow; tarsomeres III–IV and ovipositor sheath dark brown; wings yellowish, fore wing with anterior margin more fuscous; pterostigma yellow.

Male (Fig. 6). Length: 13.3 mm. Fore wing: 11.0 mm; mid-coxa evenly convex. Similar to female, antenna with 40 segments; the last flagellomere 1.5× as long as the anterior.

#### Variation.

One female with the first pair of coxa and trochanter yellowish ventrally.

#### Etymology.

The name of the species is in honor of Moacyr de Carvalho Dias, the owner of the Sitio da Ferradura.

#### Distribution.

Brazil.

*Dolichomitus moacyri* sp. n. does not seem to belong to any of Gauld et al.’s (1998) species groups. The ovipositor is only 5.0 times the length of the hind tibia; the fore wing does not have black bands, and the male is similar to the female in size and shape. In the *longicauda* species group, the male and female are similar in size and shape, but the fore wing is yellow with black bands. *Dolichomitus moacyri* sp. n. does not belong to the *taeniatus*, *irritator* or *zonatus* species groups because the male and female are not sexually dimorphic. *Dolichomitus moacyri* sp. n. is different from the species of the group *taeniatus* because it does not present a yellow dorsal stripe on the pronotum; it differs from species of the group *zonatus* because it does not present a whitish-yellow band on the flagellum of antenna. *Dolichomitus bivittatus* and *Dolichomitus hypermeces* are different in color and have longer ovipositors, at 12.0× and 21.0× as long as the hind tibia, respectively.

## Supplementary Material

XML Treatment for Dolichomitus jatai

XML Treatment for Dolichomitus moacyri
